# Transcriptome profiling of uterine leiomyosarcomas identifies a leiomyoma-like expression pattern that indicates better survival

**DOI:** 10.1038/s44276-025-00190-x

**Published:** 2025-10-29

**Authors:** Sara Khamaiseh, Riitta Koivisto-Korander, Nora Schreiber, Esa Pitkänen, Terhi Ahvenainen, Ralf Bützow, Miika Mehine, Pia Vahteristo

**Affiliations:** 1 Applied Tumor Genomics Research Program, University of Helsinki, Helsinki, Finland; 2 Department of Medical and Clinical Genetics, University of Helsinki, Helsinki, Finland; 3 iCAN Digital Precision Cancer Medicine Flagship, Helsinki, Finland; 4 Department of Obstetrics and Gynecology, Helsinki University Hospital, Helsinki, Finland; 5 Institute for Molecular Medicine Finland (FIMM), HiLIFE, University of Helsinki, Helsinki, Finland; 6 Department of Pathology, University of Helsinki and HUSLAB, Helsinki University Hospital, Helsinki, Finland

## Abstract

**Background:**

Uterine leiomyosarcomas are rare and aggressive cancers with poor survival. Their non-cancerous counterparts, uterine leiomyomas, are common tumors affecting many women during reproductive years. Distinguishing leiomyosarcomas from leiomyomas remains a diagnostic challenge. This study aimed to identify molecular biomarkers that differentiate leiomyosarcomas from leiomyomas and to assess their prognostic value.

**Methods:**

We analyzed 3′RNA sequencing data from 51 leiomyosarcomas and 44 leiomyomas with differential gene expression analysis and machine learning to identify diagnostic biomarkers. We utilized immunohistochemistry to validate the findings. We used Kaplan–Meier and Cox regression models to assess disease-specific survival in leiomyosarcoma patients.

**Results:**

Leiomyosarcomas exhibited significant dysregulation of the retinoblastoma and cell cycle pathways. Clustering based on retinoblastoma pathway genes identified a subset of leiomyosarcomas with a leiomyoma-like expression profile that associated with better survival. Machine learning-based classification identified genes with high predictive accuracy, including genes from the retinoblastoma pathway. Immunohistochemistry validated TOP2A and CDK1 as potential diagnostic biomarkers, and their higher expression was associated with worse survival. Leiomyoma driver alterations were found in 27% of leiomyosarcomas, but these showed no association with survival.

**Conclusion:**

Our results reveal molecular heterogeneity in uterine leiomyosarcomas and provide potential diagnostic and prognostic biomarkers for improved patient management.

## Introduction

Uterine leiomyosarcomas are rare and highly aggressive cancers with an estimated annual incidence of 0.4–0.9 per 100,000 women [1–3]. They are associated with poor prognosis due to high recurrence rates and a tendency to metastasize [4]. Stage is the most widely used prognostic factor, but outcomes vary considerably even within stage [5]. Symptoms such as abnormal vaginal bleeding and abdominal pain, along with morphological and imaging features, often overlap with those of non-cancerous uterine leiomyomas, complicating early diagnosis [4, 6]. Challenges in preoperative diagnostics in distinguishing leiomyomas and leiomyosarcomas may result in hidden malignancy being inadvertently disseminated during surgery [6, 7]. The primary treatment for leiomyosarcoma is hysterectomy, which is usually performed through laparotomy and often accompanied by bilateral salpingo-oophorectomy [8]. Chemotherapy is utilized for advanced or recurrent leiomyosarcoma, though a complete response is rare [8]. Molecular studies have shown that leiomyosarcomas typically display highly complex genomes and frequent alterations affecting *TP53*, *RB1*, *PTEN*, *ATRX*, and *MED12* [9–12].

Non-cancerous counterparts of leiomyosarcomas, uterine leiomyomas, are very common tumors affecting 70% of women during reproductive years [13]. One in four women with leiomyomas experience symptoms that significantly impair quality of life [14]. Typical treatment options include myomectomy or hysterectomy. Three well-established driver alterations in leiomyomas are *MED12* mutations, chromosomal rearrangements leading to HMGA2 overexpression, and biallelic mutations resulting in FH-deficiency, which together account for 80–90% of the tumors [15]. Recently, two additional rare leiomyoma subtypes have been identified: one characterized by mutations in SRCAP complex genes and another involving mutations in genes related to neddylation [16, 17]. Neddylation is a post-translational modification, where a ubiquitin-like protein, NEDD8, is attached to target proteins, affecting protein stability, activity, or localization. Other recurrent alterations, such as *COL4A5*-*COL4A6* deletions, 7q deletions, and *DEPDC5* mutations, occasionally co-occur with the aforementioned driver alterations [15, 18, 19]. Recent studies have shown that *MED12* mutations, the most common leiomyoma alterations, are associated with tumor characteristics such as tumor size and number of tumors, as well as with treatment response to gonadotropin-releasing hormone agonists and ulipristal acetate [20–24].

Leiomyoma-associated driver mutations have been reported in uterine leiomyosarcomas, suggesting that these mutations also play a role in leiomyosarcoma pathogenesis [25–27]. While *MED12* mutations can be found in up to 70–80% of leiomyomas, they are less frequent (2–30%) in leiomyosarcomas [20, 26, 28–30]. Association of leiomyosarcoma with germline *FH* mutations has been reported, although the risk appears to be lower than previously estimated [31]. Overall, leiomyoma-associated driver mutations are much rarer in leiomyosarcomas than in leiomyomas, suggesting that these tumors arise predominantly through different molecular mechanisms.

Uterine leiomyosarcomas are still primarily diagnosed by morphology, and there is no universally accepted risk stratification system in clinical use [5]. Thus, there is a critical need for robust diagnostic and prognostic markers. A limited number of studies have focused on the differential diagnosis of uterine leiomyomas and leiomyosarcomas using next-generation sequencing data [32, 33]. In this study, we applied transcriptome-wide 3′RNA sequencing to 51 uterine leiomyosarcomas and analyzed the data together with the previously reported data from 44 uterine leiomyomas and 5 myometrium control samples [34]. We aimed to identify molecular biomarkers that differentiate uterine leiomyosarcomas from leiomyomas. We also investigated whether the observed biomarkers are associated with clinical outcomes in uterine leiomyosarcoma patients.

## Materials and methods

### Patients and tissue samples

Clinical information was collected from 56 uterine leiomyosarcoma patients who had been operated on at Helsinki University Hospital during 1990–2017 (Supplementary Table 1). The median follow-up time was 64.1 months (range 1.5–372). Diagnosis was based on World Health Organization criteria [35]. The initial diagnosis was confirmed by a pathologist specialized in gynecological pathology, and in doubtful cases, a third sarcoma pathologist was consulted; all the tumors were examined by at least 2–3 pathologists. The study material for 3′RNA sequencing comprised 51 archival formalin-fixed paraffin-embedded (FFPE) uterine leiomyosarcoma tissue samples. The 3′RNA sequencing data on 44 uterine leiomyoma and 5 myometrium samples has been produced previously [34]. TOP2A and CDK1 immunohistochemistry was performed on formerly constructed tissue microarrays (TMA) that included 48 uterine leiomyosarcoma and 65 uterine leiomyoma samples [26]. Additionally, whole tissue sections from eight uterine leiomyosarcomas that were 3′RNA sequenced but not included in the TMAs were used.

### DNA extraction and Sanger sequencing

*MED12* mutation status for most uterine leiomyosarcomas and leiomyomas has been previously reported (Supplementary Table 2) [26, 34]. For the remaining samples, DNA was extracted using the conventional phenol-chloroform extraction method. Sanger sequencing of *MED12* exons 1 and 2 was performed using the Applied Biosystems ABI3730 Automatic DNA Sequencer at the Institute for Molecular Medicine Finland (FIMM). Electropherograms were visualized with Mutation Surveyor (SoftGenetics, State College, PA, USA).

### RNA extraction and 3′RNA sequencing

RNA extraction and sequencing were performed as previously described [34]. In brief, total RNA was extracted and purified from macrodissected tissue sections using the RNeasy® FFPE Kit (QIAGEN, Hilden, Germany) and deparaffinization solution (QIAGEN) according to the manufacturer’s protocol. Concentration and purity of the extracted RNA were analyzed using the LabChip GX Touch HT RNA Assay Reagent Kit (PerkinElmer, Waltham, MA, USA) and the Qubit RNA BR kit (Thermo Fisher Scientific, Waltham, MA, USA). Genomic DNA contamination was measured using the Qubit DNA BR kit (Thermo Fisher Scientific). QuantSeq 3′RNA sequencing was chosen for sequencing due to its high compatibility with FFPE tissue samples [34]. Dual-indexed mRNA libraries were prepared with QuantSeq 3′mRNA-Seq Library Prep Kit FWD (Lexogen GmbH, Vienna, Austria) according to the manufacturer’s instructions. The libraries were multiplexed and sequenced using the NovaSeq 6000 System (Illumina, San Diego, CA, USA) at FIMM with a read length of 2 × 101 base pairs and a minimum target coverage of 15 M reads for each library.

### 3′RNA sequencing data analysis

FASTQ preprocessing with default parameters was performed using the QuantSeq 3′mRNA-Seq Integrated Data Analysis Pipeline version 2.3.1 FWD UMI (Lexogen GmbH) implemented on the Bluebee® Genomics platform. In brief, the reads were trimmed using BBDuk, aligned against the Genome Reference Consortium human build 38 (GRCh38) reference genome using STAR, and counted using HTSeq. For the complete list of samples included in the 3′RNA sequencing analysis, see Supplementary Table 2. Principal component analysis and differential expression analysis were performed using DESeq2 implemented on the Chipster platform [36]. Hierarchical clustering was performed on regularized log-transformed data using Euclidean distance and the complete linkage method. The Uniform Manifold Approximation and Projection (UMAP) was created based on log10-transformed DESeq2 normalized gene expression values and using the Python package scanpy version 1.10.3 with default settings. Pathway enrichment analysis was performed using the clusterProfiler 4.0 package [37]. The false discovery rate (FDR) of 0.05 was used as a threshold for the complete list of pathways. For the most dysregulated pathways, an FDR of 0.005 was used as a cutoff, and pathways with ≤15 genes were excluded. The gene ontology enrichment analysis was performed using g:Profiler with default parameters [38].

### Machine learning-based classification

To evaluate single-gene expression data for binary classification of tumors into leiomyosarcomas and leiomyomas, we utilized the 3′RNA sequencing data from 51 leiomyosarcomas and 44 leiomyomas. The analysis was based on 1887 differentially expressed genes identified through DESeq2 analysis (Supplementary Table 3). DESeq2 normalized gene expression values were used. The predictive performance of the genes was evaluated using a logistic regression classifier and considering binary classification accuracy. The dataset was split, stratified by the tumor types, into a training set including 80% of the samples and an evaluation set with the remaining 20%. We trained a logistic regression model on the training dataset to predict the tumor type of the evaluation samples using single-gene expression data. The training and testing procedures were repeated 100 times with different data splits to obtain a robust accuracy estimate for each gene.

### Immunohistochemistry

HMGA2 and FH statuses have been previously determined for most leiomyosarcomas and leiomyomas (Supplementary Table 2) [26, 34]. Samples that were not included in the previous datasets were analyzed in this study. Immunohistochemistry was performed on TMAs for most samples, while whole tissue sections were used for eight leiomyosarcomas that were not included in the TMAs. Following deparaffinization, heat-induced antigen retrieval was carried out in a microwave oven using citrate buffer (pH 6.0). Endogenous peroxidase blocking was followed by overnight incubation with the primary antibodies. Anti-HMGA2 antibody (1:2000, 59170AP, Biocheck, South San Francisco, CA, USA) was used to evaluate HMGA2 expression levels, and a robust, indirect anti-2SC-antibody (1:500, crb2005017d, Cambridge Research Biochemicals, Billingham, UK) was used to assess the FH status [39, 40]. Expression of the two members of the retinoblastoma pathway, TOP2A and CDK1, was evaluated using anti-TOP2A (1:200, ab52934, Abcam, Cambridge, UK) and anti-CDK1 (1:200, ab133327, Abcam) antibodies. Post antibody blocking (Immunologic BV, Duiven, Netherlands: post antibody blocking for bright vision plus) was followed by incubation with a secondary poly-HRP antibody (Immunologic: Poly-HRP-GAM/R/R IgG). Expression levels were detected with the DAB Quanto (Thermo Fisher Scientific) system. A pathologist specialized in gynecological tumors (RB) evaluated the stainings using three grades (++ = strong staining, + = weak staining, and – = no staining). For HMGA2, 2SC, and CDK1, samples with strong staining were classified as positive. For TOP2A, both strong and weak stainings were considered positive.

### Statistical analyses

Statistical analyses were performed using R version 4.4.0 (www.R-project.org). Survival analyses were conducted using the survival 3.7.0 and survminer 0.5.0 packages. Univariate Cox proportional hazards regression models were applied to both categorical and continuous variables. Kaplan–Meier curves were used to illustrate survival distributions across categorical variables. Regularized log-transformed 3′RNA expression values of *TOP2A* and *CDK1* were analyzed as continuous variables. 3′RNA expression levels were also categorized into high and low groups based on the optimal cutoff value. Multivariate Cox proportional hazards regression models were adjusted for age at diagnosis and stage (I versus II–IV). Disease-specific survival was calculated from the date of diagnosis to the date of death due to the disease or the last available follow-up. Patients who were alive at the last follow-up or died from other causes were censored. The Fisher’s exact test and Pearson’s chi-square test were used for comparing categorical variables. All statistical tests were two-sided, and *P*-values < 0.05 were considered statistically significant.

## Results

### Differential expression analysis reveals dysregulation of the retinoblastoma and cell cycle pathways in uterine leiomyosarcomas

**Fig. 1:**
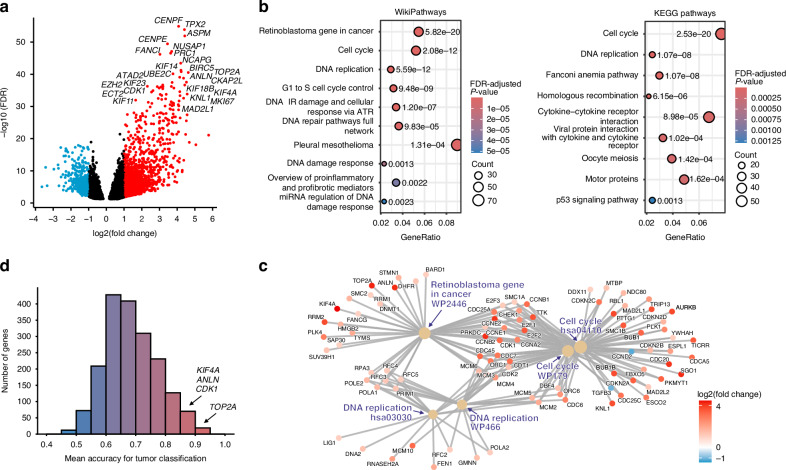
Differential gene expression analysis, pathway analysis, and tumor classification. **a** Differentially expressed genes in uterine leiomyosarcomas compared to uterine leiomyomas and myometrium. The 1887 differentially expressed genes are shown with overexpressed genes in red and downregulated genes in blue. The 25 most significantly overexpressed genes are highlighted. **b** Dysregulated pathways identified through enrichment analyses using WikiPathway and KEGG pathways and a false discovery rate (FDR) cutoff of 0.005. The most significantly enriched pathways included the retinoblastoma gene in cancer, cell cycle, and DNA replication pathways. **c** Network plot illustrating the associations between the most significantly enriched pathways from two independent enrichment analyses (WikiPathways and KEGG). This association is determined through shared genes that are differentially expressed in leiomyosarcomas compared to leiomyomas and myometrium. **d** Predictive accuracy of differentially expressed genes in classifying uterine leiomyosarcomas and uterine leiomyomas. The figure illustrates the distribution of mean accuracy values for single-gene classification based on 100 prediction attempts. Highlighted genes demonstrate high predictive accuracy (≥85%), rank among the 25 most differentially expressed genes, and are involved in the retinoblastoma pathway.

Gene expression profiles of 51 leiomyosarcomas were analyzed together with a previously published dataset of 44 leiomyomas and 5 myometrium samples [34]. Differential expression analysis comparing leiomyosarcomas against leiomyomas and myometrium samples revealed 1887 differentially expressed genes (|fold change| >2; FDR < 0.05, Fig. 1a, Supplementary Table 3). Most of these genes were overexpressed (1500/1887; 79%) while only one-fifth (387/1887; 21%) was downregulated. The 25 most differentially expressed genes were all overexpressed (Fig. 1a).

Pathway enrichment analysis with two independent tools revealed retinoblastoma gene in cancer and cell cycle (WikiPathways) and cell cycle and DNA replication (KEGG) as the most significantly altered pathways in leiomyosarcomas compared to leiomyomas and myometrium (Fig. 1b). Notably, there is a substantial overlap between the genes involved in the retinoblastoma pathway and those in the cell cycle and DNA replication pathways, emphasizing the relationship between the enriched pathways (Fig. 1c). See Supplementary Table 4 for the full list of dysregulated pathways. In addition, gene ontology analysis identified significantly enriched biological processes, including cell cycle process, humoral immune response, and smooth muscle contraction (Supplementary Table 5).

### Machine learning classification identifies genes with high predictive accuracy for distinguishing uterine leiomyosarcomas from uterine leiomyomas

We evaluated single-gene expression data of the 1887 differentially expressed genes to assess their predictive accuracy in distinguishing leiomyosarcomas from leiomyomas (Fig. 1d). Logistic regression prediction analysis revealed that 89 genes achieved a mean classification accuracy of at least 85% (Supplementary Table 6). Twelve percent (11/89) of the genes with ≥85% classification accuracy were members of the retinoblastoma pathway, of which four —*TOP2A*, *ANLN*, *KIF4A*, and *CDK1*— were also among the 25 most differentially expressed genes (Fig. 1d).

### Uterine leiomyosarcomas with expression profiles similar to uterine leiomyomas are associated with improved survival

**Fig. 2:**
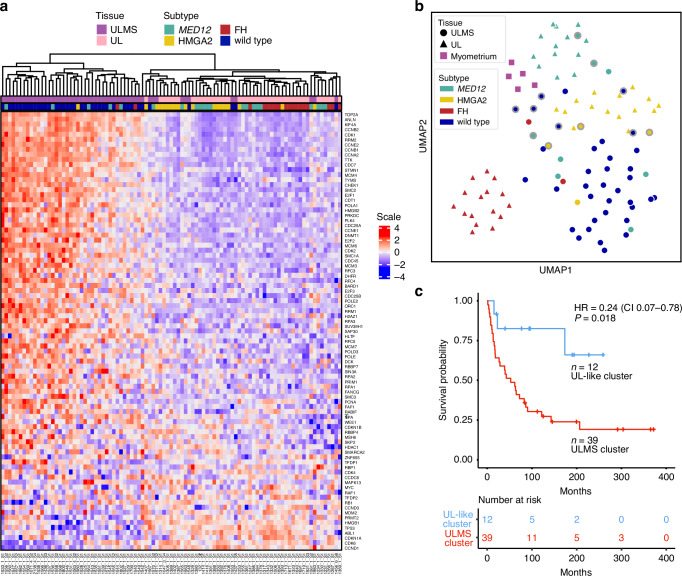
Distinct gene expression profiles in uterine leiomyosarcomas and uterine leiomyomas. **a** Hierarchical clustering analysis of 51 uterine leiomyosarcomas and 44 uterine leiomyomas using genes of the retinoblastoma signaling pathway (*n* = 87). Twelve leiomyosarcomas clustered together with leiomyomas, and half of them (6/12; 50%) harbored a leiomyoma driver mutation. **b** UMAP visualization of whole transcriptome data revealed that leiomyosarcomas display a more heterogeneous gene expression profile compared to leiomyomas, which group according to their molecular driver alteration. Twelve leiomyosarcomas that clustered with leiomyomas based on genes of the retinoblastoma pathway are highlighted with outer gray borders. **c** Kaplan–Meier curve comparing disease-specific survival between patients, whose uterine leiomyosarcomas clustered with leiomyomas (based on the retinoblastoma pathway genes) and those whose leiomyosarcomas did not. Univariate Cox regression revealed that patients with leiomyoma-like leiomyosarcomas had better disease-specific survival. ULMS uterine leiomyosarcoma, UL uterine leiomyoma, HR hazard ratio, 95% CI confidence interval.

We next performed hierarchical clustering analysis using the genes of the retinoblastoma pathway (*n* = 87) (Fig. 2a). Interestingly, a subset of leiomyosarcomas (12/51; 24%) clustered together with leiomyomas. These 12 leiomyosarcomas were also grouped with leiomyomas in the UMAP visualization based on the whole transcriptome (highlighted with outer gray borders in Fig. 2b). In the principal component analysis (Supplementary Fig. 1), the same 12 leiomyosarcomas grouped near the leiomyomas. Histopathological re-evaluation was conducted for these 12 samples (Supplementary Table 7), and all were confirmed as leiomyosarcomas according to the World Health Organization criteria [35].

We then investigated whether the observed clustering pattern was associated with disease-specific survival. Indeed, patients whose leiomyosarcomas exhibited a leiomyoma-like expression pattern had better survival (hazard ratio [HR] 0.24; confidence interval [CI] 0.07–0.78; *P* = 0.018) (Fig. 2c). Beyond survival, the clustering groups showed no statistically significant differences in clinical characteristics listed in Supplementary Table 8.

We assessed the association of age, stage, and tumor size with clinical outcomes. All three variables were significantly associated with worse disease-specific survival (Table 1). Given the number of events (*n* = 33), our multivariate analysis was restricted to three variables: age, stage, and leiomyoma-like cluster. Tumor size was excluded due to missing data in a substantial proportion of cases (14/56). After adjusting for age and stage, the clustering pattern showed marginal significance as an independent prognostic factor (HR 0.33; CI 0.10–1.12; *P* = 0.076). Only stage I tumors were sufficient for subgroup analysis, in which the previously defined clustering pattern was significantly associated with survival (HR 0.12; CI 0.02–0.90; *P* = 0.039) (Supplementary Fig. 2a).

**Table 1:** Univariate and multivariate analyses of clinical variables and leiomyoma-like clustering as prognostic factors.

	Univariate analysis		Multivariate analysis	
	HR (95% CI)	*P*-value	HR (95% CI)	*P*-value
Age at diagnosis (y)	1.07 (1.04, 1.10)	**<0.001**	1.07 (1.02–1.11)	**0.001**
Stage (I vs. II–IV)	3.29 (1.62, 6.67)	**<0.001**	2.37 (1.11–5.06)	**0.026**
Primary tumor size (cm)^a^	1.08 (1.03, 1.13)	**0.003**		
Leiomyoma-like cluster	0.24 (0.07, 0.78)	**0.018**	0.33 (0.10–1.12)	0.076

*P*-values < 0.05 were considered statistically significant (in bold).

*HR* hazard ratio, *95% CI* confidence interval.

^a^Tumor size was excluded from the multivariate analysis due to missing data in a substantial proportion of cases (14/51).

### Immunohistochemistry demonstrates the diagnostic potential of TOP2A and CDK1

**Fig. 3:**
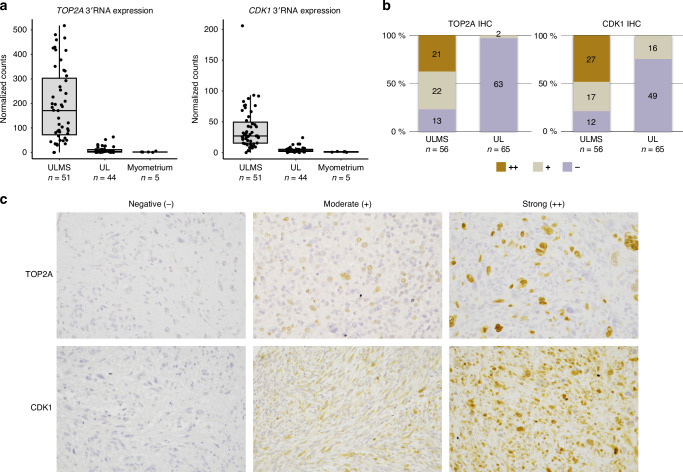
Immunohistochemistry confirms increased expression of TOP2A and CDK1 in uterine leiomyosarcomas. **a** Overexpression of *TOP2A* and *CDK1* in uterine leiomyosarcomas based on 3′RNA expression data. Box plots show the median and the first and third quartiles. Whiskers extend to 1.5 times the interquartile range beyond the quartiles. **b** Immunohistochemistry staining results of TOP2A and CDK1 in uterine leiomyosarcomas and uterine leiomyomas. **c** Representative images on TOP2A and CDK1 immunostainings in uterine leiomyosarcomas. ULMS uterine leiomyosarcoma, UL uterine leiomyoma, IHC immunohistochemistry.

We then assessed the protein level expression of TOP2A and CDK1, two members of the retinoblastoma pathway, utilizing immunohistochemistry. Both *TOP2A* and *CDK1* were among the top 25 differentially expressed genes (Fig. 1a) and were overexpressed in leiomyosarcomas (Fig. 3a). They also showed high predictive accuracy in machine learning-based classification (Fig. 1d). Protein expression levels were evaluated in 56 leiomyosarcomas and 65 leiomyomas. Immunohistochemistry results are presented in Fig. 3b, and representative images of strong (++), moderate (+), and negative (−) stainings are shown in Fig. 3c.

A significant proportion of leiomyosarcomas exhibited high or moderate TOP2A protein expression (43/56; 77%). This contrasted with leiomyomas, where low expression was predominant (63/65; 97%) (Fig. 3b). There was a significant difference in TOP2A protein expression between leiomyosarcomas and leiomyomas across the three staining intensities (Pearson’s chi-square test *P* = 5.479e-16). Similarly, a considerable proportion of leiomyosarcomas showed high or moderate CDK1 protein expression (44/56; 79%), while most leiomyomas displayed low expression (49/65; 75%) (Fig. 3b). A significant difference was also observed for CDK1 across the three staining intensities (Pearson’s chi-square test *P* = 2.205e-11). For TOP2A, both strong (++) and moderate (+) staining were considered positive, resulting in 77% sensitivity and 97% specificity. For CDK1, only strong (++) staining was classified as positive to avoid false positives, as moderate staining was observed in 25% (16/65) of leiomyomas. This classification resulted in 48% sensitivity and 100% specificity.

### Increased TOP2A and CDK1 expression predicts poor survival

**Fig. 4:**
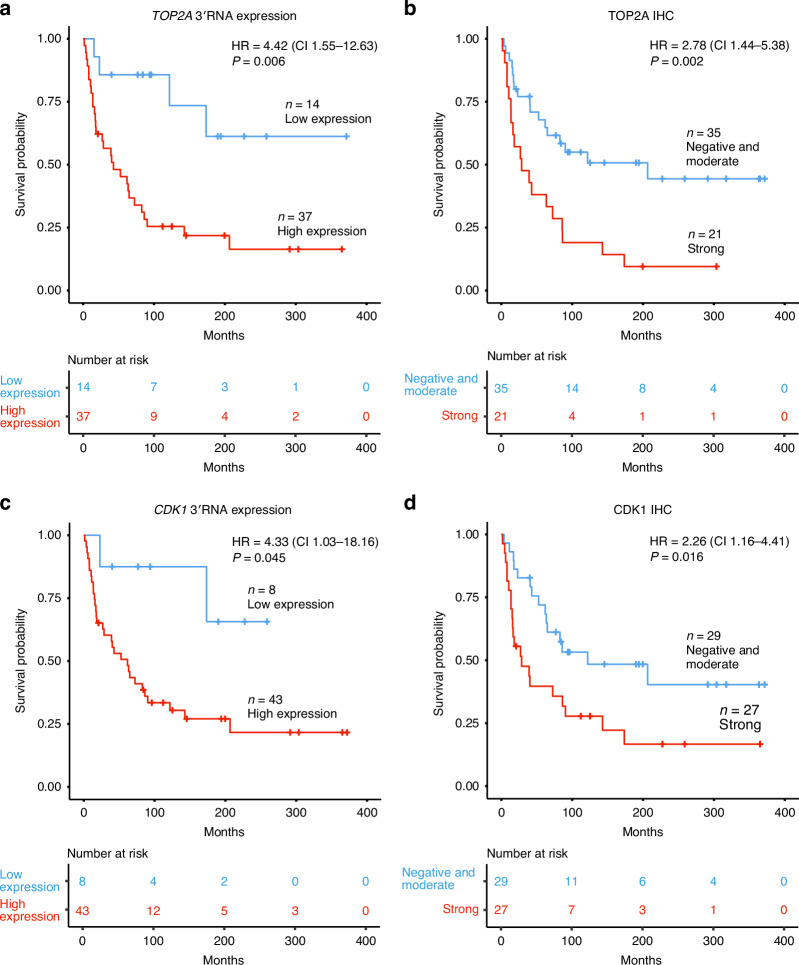
Association of TOP2A and CDK1 expression with disease-specific survival. Kaplan–Meier survival curve and univariate Cox regression analyses comparing disease-specific survival between patients with **a** high and low *TOP2A* 3′RNA expression levels defined by the optimal cutoff, **b** TOP2A protein expression levels based on immunohistochemistry, **c** high and low *CDK1* 3′RNA expression levels defined by the optimal cutoff, and **d** CDK1 protein expression levels based on immunohistochemistry. IHC immunohistochemistry, HR hazard ratio, 95% CI confidence interval.

To assess the prognostic relevance of TOP2A and CDK1, we analyzed their association with disease-specific survival. High *TOP2A* 3′RNA expression determined by the optimal cutoff was associated with worse survival (HR 4.42; CI 1.55–12.63; *P* = 0.006) (Fig. 4a). When analyzed as a continuous variable, higher *TOP2A* 3′RNA expression was also correlated with worse survival (HR 1.39; CI 1.02–1.90; *P* = 0.039). This association remained significant in multivariate analysis after adjusting for age and stage (Table 2). No difference in survival was observed between patients with moderate or negative staining for both TOP2A and CDK1 in immunohistochemistry (Supplementary Table 9). Therefore, these groups were combined as the reference category and compared to patients with strong staining. High TOP2A protein expression was associated with worse survival (HR 2.78; CI 1.44–5.38; *P* = 0.002) (Fig. 4b), and this association remained significant in multivariate analysis (Table 2). In stage I leiomyosarcomas, higher TOP2A expression was associated with worse survival at both the RNA and protein levels (Supplementary Table 10 and Supplementary Fig. 2b, c).

**Table 2:** Univariate and multivariate analyses of TOP2A and CDK1 expression as prognostic markers.

Univariate analysis			Multivariate analysis		
	HR (95% CI)	*P*-value		HR (95% CI)	*P*-value
***TOP2A*** **3′RNA**	1.39 (1.02–1.90)	**0.039**	***TOP2A*** **3′RNA**	1.44 (1.00–2.08)	**0.048**
			age at diagnosis	1.07 (1.03–1.12)	<0.001
			stage	3.08 (1.47–6.48)	0.003
**TOP2A IHC**	2.78 (1.44–5.38)	**0.002**	**TOP2A IHC**	2.35 (1.10–4.99)	**0.027**
			age at diagnosis	1.06 (1.02, 1.10)	0.001
			stage	3.21 (1.56–6.61)	0.002
***CDK1*** **3′RNA**	1.28 (0.89–1.86)	0.182	***CDK1*** **3′RNA**	1.41 (0.91–2.18)	0.126
			age at diagnosis	1.07 (1.03–1.12)	<0.001
			stage	2.77 (1.31–5.84)	0.008
**CDK1 IHC**	2.26 (1.16–4.41)	**0.016**	**CDK1 IHC**	2.49 (1.20–5.14)	**0.014**
			age at diagnosis	1.08 (1.04–1.12)	<0.001
			stage	2.84 (1.38–5.84)	0.005

*P*-values in bold indicate markers that remained independent prognostic factors after adjusting for age and stage (*P* < 0.05). Regularized log-transformed 3′RNA expression values were treated as a continuous variable.

*HR* hazard ratio, *95% CI* confidence interval, *IHC* immunohistochemistry.

Similarly, high *CDK1* 3′RNA expression determined by the optimal cutoff was associated with worse disease-specific survival (HR 4.33; CI 1.03–18.16; *P* = 0.045) (Fig. 4c). However, when analyzed as a continuous variable, *CDK1* 3′RNA expression did not reach statistical significance (HR 1.28; CI 0.89–1.86; *P* = 0.182). High CDK1 protein expression was associated with worse survival (HR 2.26; CI 1.16–4.41; *P* = 0.016) (Fig. 4d), and this association remained significant in multivariate analysis (Table 2). In stage I tumors, we did not find an association between CDK1 RNA or protein expression levels and survival (Supplementary Table 10 and Supplementary Fig. 2d, e). We also did not identify any associations between TOP2A or CDK1 levels and clinical characteristics other than survival (Supplementary Table 11).

### Leiomyoma driver mutations do not predict survival in uterine leiomyosarcoma patients

Leiomyoma driver mutation status was available for all 51 leiomyosarcomas included in the 3′RNA expression analysis. Fourteen (14/51; 27%) tumors harbored a mutation: eight were *MED12* positive, four displayed HMGA2 overexpression, and two exhibited FH-deficiency (Supplementary Table 2) [26]. Among the 12 leiomyosarcomas that clustered with leiomyomas based on retinoblastoma pathway genes, 50% (6/12) harbored a leiomyoma driver mutation, compared to 21% (8/39) in the leiomyosarcoma cluster (Fisher’s exact test *P* = 0.066) (Fig. 2a). However, we did not find significant association between the leiomyoma mutation status in leiomyosarcomas and disease-specific survival (HR 1.20; CI 0.56–2.56; *P* = 0.644). Leiomyosarcomas did not cluster together based on the presence of the established leiomyoma-associated driver mutations, unlike leiomyomas, which clustered according to the underlying *MED12*, HMGA2, or FH alteration (Fig. 2b and Supplementary Fig. 1).

### Increased *IRS4* expression and *COL4A5-COL4A6* deletions in a subset of uterine leiomyosarcomas

We observed elevated *IRS4* expression in 5 out of 51 (10%) leiomyosarcomas (Supplementary Fig. 3a). *IRS4* overexpression has been previously observed in leiomyomas harboring *COL4A5*-*COL4A6* deletions [15]. Exome sequencing data was available from two leiomyosarcomas that showed elevated *IRS4* expression (1553 and 1556) [9], and the data confirmed a *COL4A5-COL4A6* deletion in both samples (Supplementary Fig. 3b).

To further assess the *IRS4* expression in uterine leiomyosarcomas, we utilized a publicly available TCGA dataset including RNA and exome sequencing data from 27 uterine leiomyosarcomas [41, 42]. Gene expression profiling revealed high *IRS4* expression in 4/27 (15%) leiomyosarcomas (Supplementary Fig. 3a). We identified a *COL4A5-COL4A6* deletion in three out of four of these tumors, while all samples with normal *IRS4* expression were negative for these deletions (Fisher’s exact test *P* = 0.001) (Supplementary Fig. 3b).

## Discussion

Distinguishing between uterine leiomyosarcomas and leiomyomas remains a significant diagnostic challenge. Current diagnostic methods rely on clinical symptoms, imaging characteristics, and morphology, all of which can overlap between leiomyomas and leiomyosarcomas [4]. This study aimed to identify molecular biomarkers that differentiate leiomyosarcomas from leiomyomas using 3′RNA sequencing. Differential gene expression and functional pathway analyses revealed dysregulation of the cell cycle and DNA replication pathways in leiomyosarcomas. This is in line with the previous studies that have highlighted the involvement of cell cycle-associated processes in leiomyosarcoma pathogenesis [33, 43]. Notably, the retinoblastoma pathway emerged as the most significantly altered pathway in WikiPathways. Machine learning classification based on single-gene expression data identified a subset of differentially expressed genes with high accuracy in distinguishing leiomyosarcomas from leiomyomas. This subset included several retinoblastoma pathway genes, but did not include *TP53* and *RB1*, which are among the most frequently mutated genes in uterine leiomyosarcomas [9–12]. Previous immunohistochemistry studies have tested p53 and Rb as potential biomarkers to differentiate leiomyosarcomas from leiomyomas, but single markers were insufficient, leading to recommendations for using a multi-marker immunohistochemistry panel [44, 45]. In this study, we evaluated TOP2A and CDK1—two most differentially expressed genes within the retinoblastoma pathway that also demonstrated high predictive accuracy in machine learning-based classification— as potential biomarkers using immunohistochemistry.

Previous array-based expression studies have shown that *TOP2A* and *CDK1* are overexpressed in leiomyosarcomas when compared to leiomyomas [46, 47]. Our findings using 3′RNA sequencing support these observations. *TOP2A* encodes a DNA topoisomerase enzyme that is crucial in regulating DNA topology during transcription, replication, and cell division. To date, only two studies have demonstrated TOP2A overexpression in leiomyosarcomas versus leiomyomas through immunohistochemistry [47, 48]. CDK1, a key regulator of the cell cycle, controls the transition from the G2 phase to mitosis —a process often dysregulated in cancer. Although no previous studies have analyzed CDK1 overexpression in leiomyosarcomas versus leiomyomas through immunohistochemistry, CDK1 has been proposed as a potential diagnostic biomarker for rhabdomyosarcoma [49]. Immunohistochemistry results indicated high specificity for both TOP2A and CDK1, with greater sensitivity for TOP2A. The machine learning classification results supported these findings, with *TOP2A* demonstrating slightly higher predictive accuracy. These findings indicate TOP2A and CDK1 as potential diagnostic biomarkers for distinguishing leiomyosarcomas from leiomyomas.

Clinical outcome in uterine leiomyosarcoma patients varies widely. Even when accounting for the known prognostic factors like age and stage, determining the likely disease course remains challenging [5]. Molecular characterization of tumors may provide significant prognostic insights. Previous studies that used only uterine leiomyosarcoma samples have identified molecular subtypes that were associated with varying treatment responses and clinical outcomes [50–52]. In this study, we analyzed both uterine leiomyosarcomas and leiomyomas and found that a leiomyoma-like expression profile in leiomyosarcomas is associated with better disease-specific survival. After adjusting for age and stage in multivariate analysis, the leiomyoma-like clustering pattern reached marginal significance as an independent prognostic factor. These findings provide novel insight that could assist clinical decision-making.

We subsequently evaluated whether TOP2A and CDK1 expression are associated with clinical outcome. Notably, high expression of both TOP2A and CDK1 independently predicted worse survival in uterine leiomyosarcoma patients at all stages. High expression of TOP2A has been correlated with poor prognosis in various cancers, including breast, prostate, and ovarian malignancies [53, 54]. To our knowledge, the study by Baiocchi et al. is the only study to date that has investigated the prognostic value of TOP2A protein expression in uterine leiomyosarcomas [48]. They found that TOP2A was highly expressed in tumors with a high mitotic index and advanced-stage disease, but the expression did not correlate with survival. Unlike their study, we found no association with TOP2A level and tumor stage. Several methodological differences may account for the discrepancy in findings, including a larger sample size and a median follow-up period three times longer in this study, and a different scoring and categorization approach between the studies. CDK1 expression has not been previously assessed as a prognostic indicator in uterine leiomyosarcomas. However, it has been identified as a potential prognostic biomarker in other cancers, for instance, in lung adenocarcinoma, where elevated CDK1 expression has been associated with more advanced stage, poor tumor differentiation, and the presence of *TP53* mutations [55]. Our results indicate both TOP2A and CDK1 as potential prognostic biomarkers in uterine leiomyosarcomas.

Leiomyoma-associated driver alterations in *MED12*, HMGA2, and FH have been reported in up to one-third of uterine leiomyosarcomas [25–27, 29, 30]. In this study, half of the leiomyoma-like leiomyosarcomas with better disease-specific survival harbored a leiomyoma driver mutation, but no significant association was found between the mutation status and survival. While leiomyomas clustered distinctly according to driver mutation status, leiomyosarcomas did not. Most likely, the clustering patterns reflect the relative stability of leiomyoma genomes, opposed to the much larger number of alterations in leiomyosarcomas. In leiomyomas, additional genetic alterations, including *COL4A5*-*COL4A6* deletions, may co-occur with the primary driver mutations [15, 18]. This is the first study to report *COL4A5-COL4A6* deletions in uterine leiomyosarcomas, suggesting that these mutations may contribute to leiomyosarcoma pathogenesis.

Limitations of the study include a relatively small sample size, which is due to the rarity of uterine leiomyosarcomas. Thus, further studies with independent sample series are required to validate these findings and to determine their clinical applicability. Also, primary and metastatic tumors should ideally be analyzed separately. Here, a few metastatic samples were included as they were the only available samples from some patients.

Molecular tumor characteristics can provide valuable assistance in clinical decision-making. In this study, tumor stratification based on retinoblastoma pathway genes revealed that some uterine leiomyosarcomas exhibit a leiomyoma-like expression profile in leiomyosarcomas and that this profile is associated with better disease-specific survival. Two members of the retinoblastoma pathway, TOP2A and CDK1, were further shown as potential diagnostic biomarkers to distinguish leiomyomas from leiomyosarcomas and as prognostic tools in leiomyosarcoma patients.

## Supplementary information

Supplementary Information

Supplementary Information

## Data Availability

All data relevant to this study are included within the article or provided in the supplementary material. The raw data are not publicly available due to compliance with the ethics approval and confidentiality agreements.
