# Supplementary material for: Transcriptome profiling of uterine leiomyosarcomas identifies a leiomyoma-like expression pattern that indicates better survival
Source: BJC Rep. 2025 Oct 29;3:76. doi: 10.1038/s44276-025-00190-x (PMC12572308; doi:10.1038/s44276-025-00190-x)
Supplement: Supplementary file 1 — Supplementary Information [file 44276_2025_190_MOESM1_ESM.docx]

**Transcriptome profiling of uterine leiomyosarcomas identifies a leiomyoma-like expression pattern that indicates better survival**

Sara Khamaiseh, Riitta Koivisto-Korander, Nora Schreiber, Esa Pitkänen, Terhi Ahvenainen, Ralf Bützow, Miika Mehine, and Pia Vahteristo

**Supplementary figures**

**
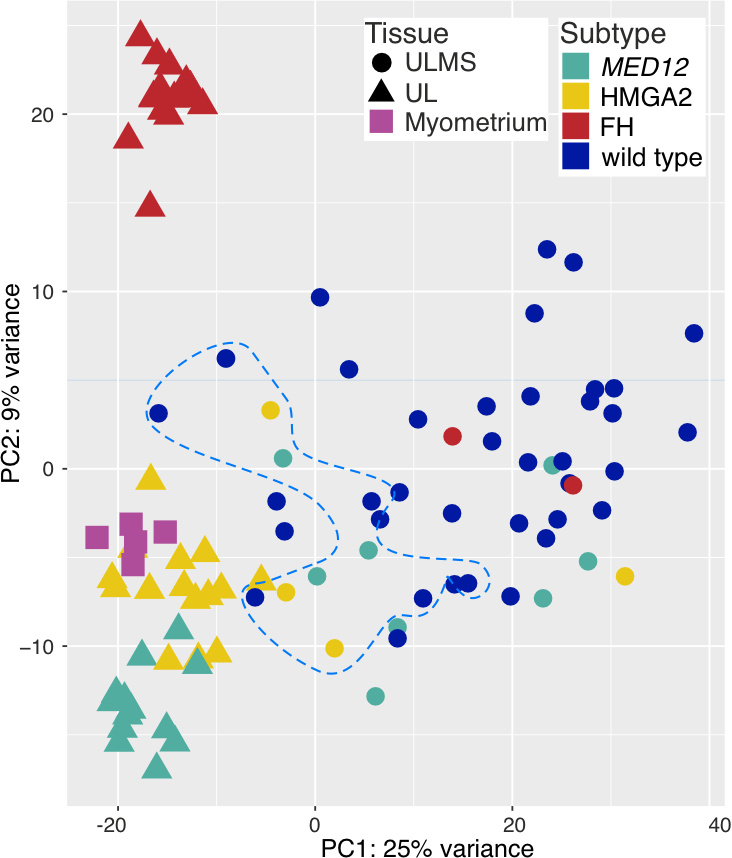
**

**Supplementary Fig. 1** Principal component analysis of whole transcriptome profiles of uterine leiomyosarcoma, leiomyoma, and myometrium samples. The analysis demonstrates that leiomyosarcomas exhibit a more heterogeneous gene expression profile compared to leiomyomas, which cluster according to their molecular driver alteration. Twelve leiomyosarcomas that clustered with leiomyomas based on genes of the retinoblastoma pathway are highlighted by a blue dashed line. ULMS, uterine leiomyosarcoma; UL, uterine leiomyoma.

| 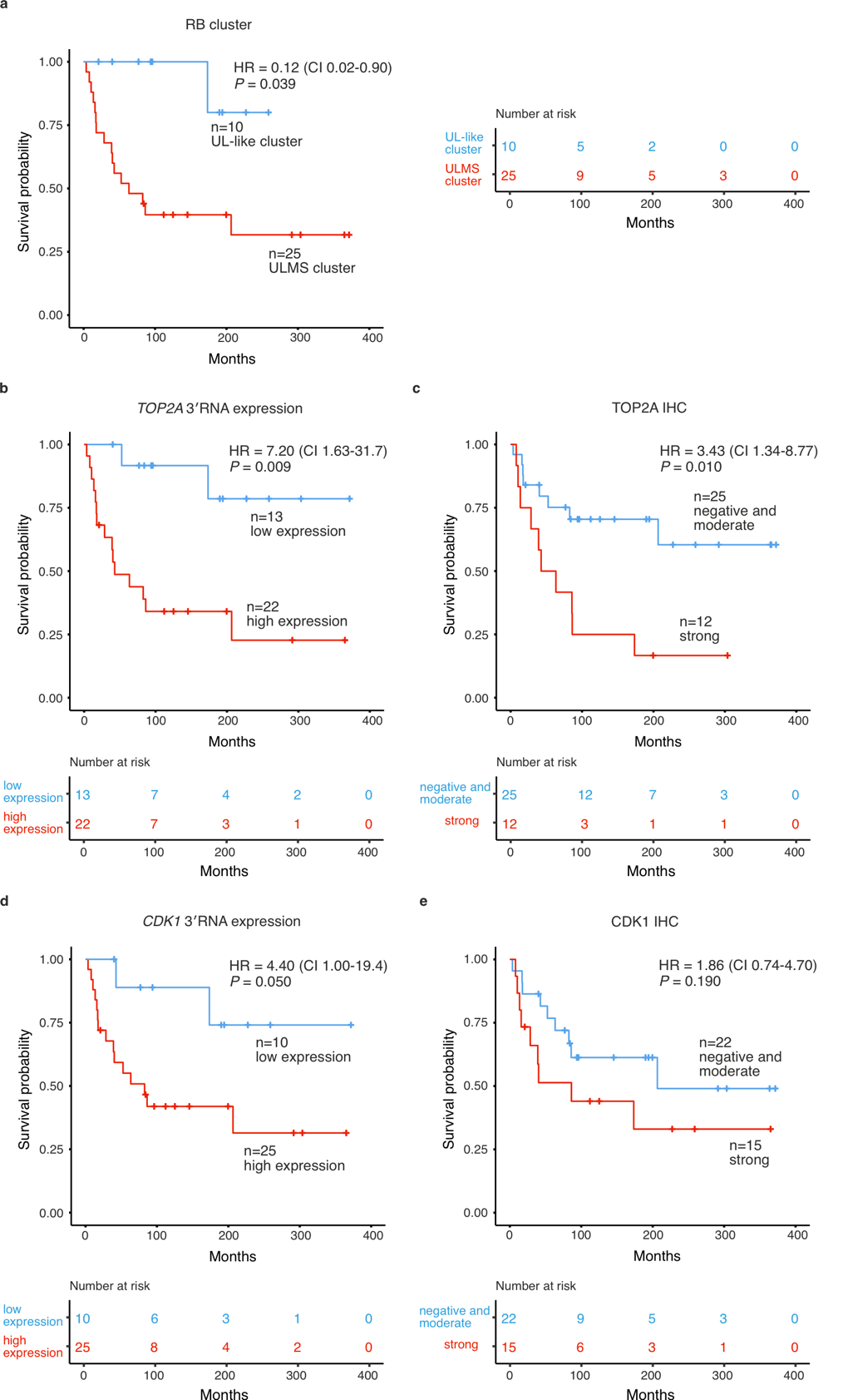 |
| --- |
| **Supplementary Fig. 2** Kaplan-Meier curves and univariate Cox regression analyses comparing disease-specific survival of stage I uterine leiomyosarcoma patients based on, **a** leiomyoma-like expression clustering feature defined by the retinoblastoma pathway, **b** *TOP2A* 3′RNA expression levels determined by the optimal cutoff, **c** TOP2A protein expression levels based on immunohistochemistry, **d** *CDK1* 3′RNA expression levels determined by the optimal cutoff, and **e** CDK1 protein expression levels based on immunohistochemistry. ULMS, uterine leiomyosarcoma; UL, uterine leiomyoma; IHC, immunohistochemistry; HR, hazard ratio; 95% CI, confidence interval. |

**
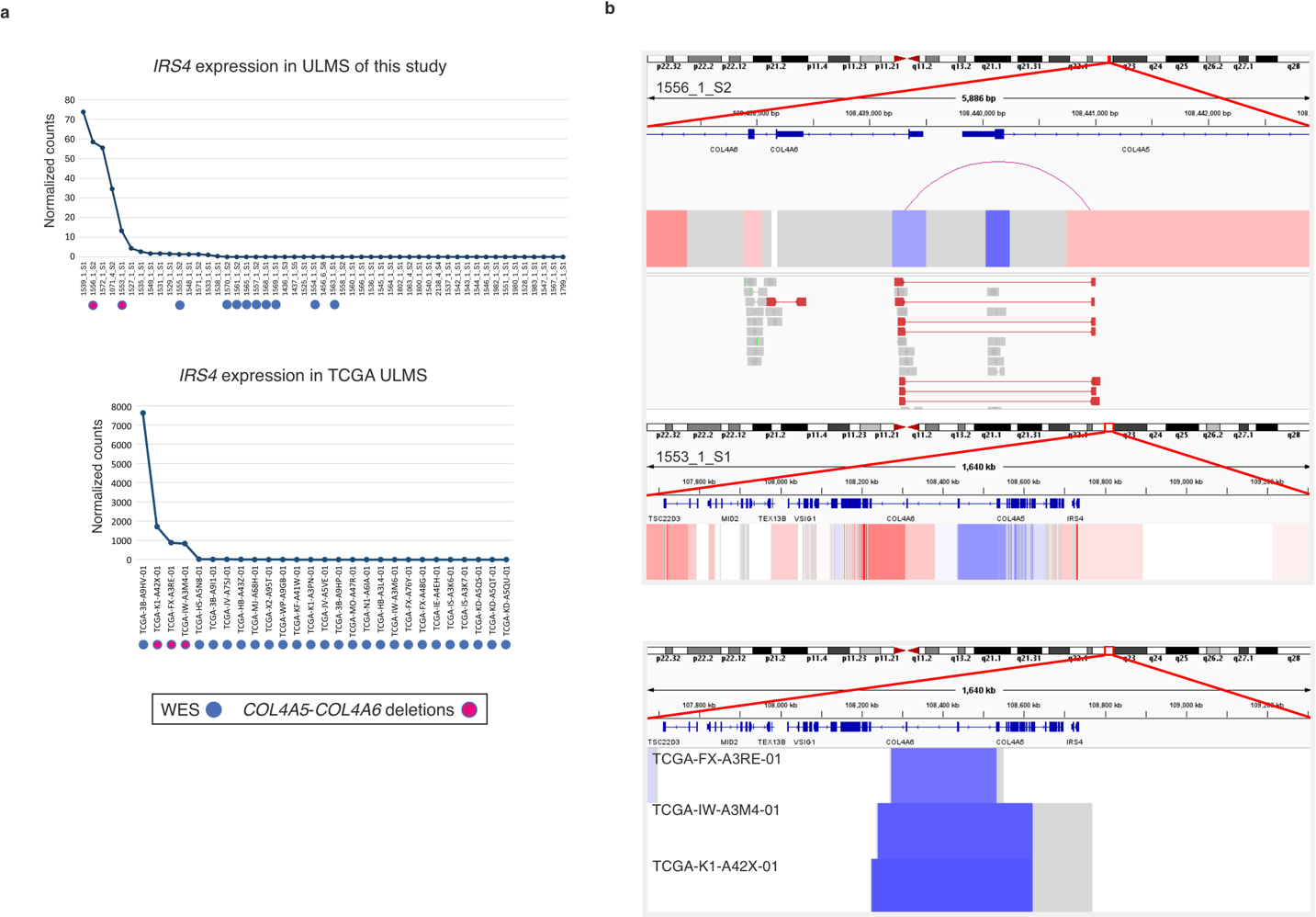
**

**Supplementary Fig. 3** *IRS4* overexpression and *COL4A5-COL4A6* deletions in a subset of uterine leiomyosarcomas. **a** Significant overexpression of *IRS4* in five leiomyosarcomas of this study and four leiomyosarcomas from TCGA data. Overexpression was defined as an increase of more than two times the average *IRS4* expression across all samples. **b** *COL4A5-COL4A6* deletions were identified in two leiomyosarcomas from this study and three leiomyosarcomas from the TCGA data, all of which exhibited *IRS4* overexpression. ULMS, uterine leiomyosarcoma; WES, whole exome sequencing.
